# Calcification of the intervertebral disc and ossification of posterior longitudinal ligament in children

**DOI:** 10.1186/s12891-018-2227-z

**Published:** 2018-09-05

**Authors:** Jun-Jie Du, Yu-Fei Chen, Ye Peng, Xiao-jie Li, Wei Ma

**Affiliations:** Department of Orthopaedics, Air Force General Hospital of PLA, 30 Fucheng Road, Beijing, 100142 People’s Republic of China

**Keywords:** Intervertebral disc calcification, Ossification of the posterior longitudinal ligament, Pediatric, Cervical spine

## Abstract

**Background:**

IDC in children, first reported by Baron in 1924, is very rare. OPLL of the cervical spine mainly affect people ages 50–70 years. The coexistence of IDC and OPLL in children is very rare, only six cases with 3 to 24 months’ follow-up were reported to date.

**Case presentation:**

A 6-year-old boy presented with complains of neck pain at July 2007. The boy was treated by conservative treatment and observed up for 9 years. Neck pain greatly improved after a one-month conservative treatment and never recur. Laboratory tests revealed elevated ESR and CRP at admission and found nothing abnormal at 19-month and 9-year follow-up. Computed tomography and magnetic resonance imaging revealed IDC at C2/3, C3/4 and OPLL at C3/4 at admission and found minor calcification at C2/3 remained but calcification at C3/4 and OPLL at C3/4 completely disappeared at 19-month and 9-year follow-up. Nineteen months after initial diagnosis, restoration of T2-weighted signal intensity of C2/3 and C3/4 discs was observed through MRI. Loss of T2-weighted signal intensity of C2/3 disc and decrease of T2-weighted signal intensity of C3/4 disc was observed at 9-year follow-up.

**Conclusions:**

IDC with OPLL in children is very rare. Conservative treatments are recommended with affirmative short-term and long-term clinical effects. More intensive observation with long-term follow-ups may be needed to warrant the clinical effects.

## Background

Calcification of intervertebral disc in children is rare. Since firstly reported by Baron in 1924, approximately 400 cases were reported [1]. Although traumatic, infectious, inflammatory, and nutritional mechanisms were thought to contribute to calcification of intervertebral disc in children, the detailed etiology remain not defined. Ossification of the posterior longitudinal ligament (OPLL) mainly affect people ages 50–70 years, also with unclear etiology. Calcification of intervertebral disc in children is usually thought to be self-limiting with favorable prognosis, while OPLL in adults usually aggravates gradually and needs surgery when present with myelopathy or radiculopathy. The coexistence of calcification of intervertebral disc and OPLL is very rare, only six cases with 3 to 24 months’ follow-up were reported to date [2–6]. We reported the first two cases of cervical intervertebral disc calcification combined with OPLL in children in 2012 [3] and followed one case for more than nine years. The purpose of this case report is to describe the 9-year follow-up result. To our knowledge, long-term follow-up for cervical intervertebral disc calcification combined with OPLL is firstly reported here.

## Case presentation

**Fig. 1:**
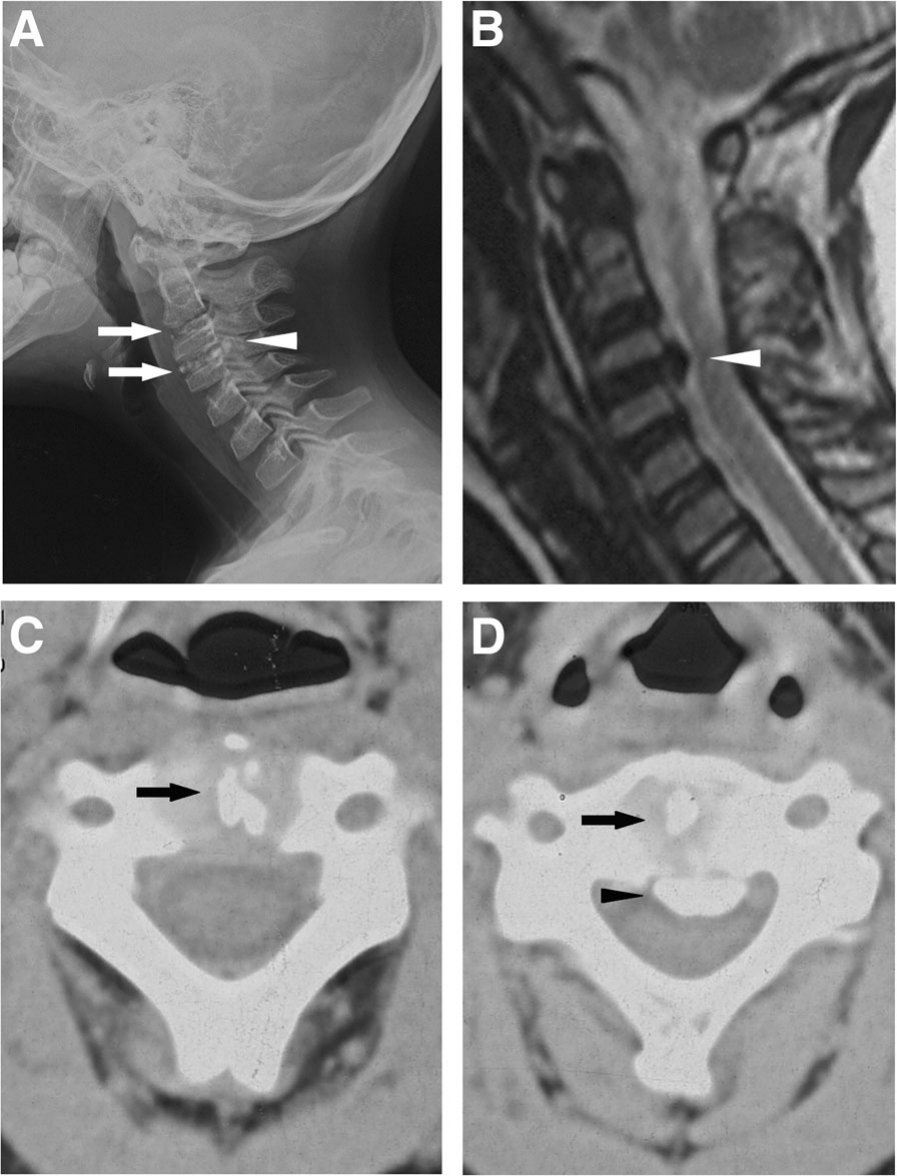
Radiological imaging at admission. **a** Lateral cervical spine radiograph showed C2/3and C3/4 IDC (thin arrows) and OPLL at C3 and C4 (thick arrow). **b** Magnetic resonance imaging showed that spinal cord was compressed anteriorly at the C3/4 level (arrow). **c** Axial computed tomography through C2/3 revealed IDC (thick arrows) at C2/3. **d** Axial computed tomography through C3/4 revealed IDC (thick arrows) at C3/4 and OPLL at C3/4 level (thin arrow). (Adopted and reedited from Du et al. [3] with permissions of all authors)

A 6-year-old boy presented with right-sided neck pain for 6 months was admitted in our institution on July 2007, with no history of recent trauma, fever or infection. The pain localized in the right side of neck, without radiating pain. The pain exacerbated for several days and not alleviated by using analgesics. Visual Analogue Scale (VAS) for cervical pain was 7.0. Physical examination revealed no palpable masses or torticollis. Neurological examination revealed nothing abnormal. Laboratory tests revealed normal white blood cell count (6170/mm^3^, normal range: 5000–12,000/mm^3^) and elevated ESR (69 mm/h, normal range: 0 to 20 mm/h) and CRP (11.80 mg/L, normal range: 0 to 5 mg/L). Radiograph and CT showed calcification of intervertebral disc at C2/3 and C3/4 levels, accompanied by C3/4 level OPLL (Fig. 1a, c and d). MRI revealed decreased signal intensity of C2–4 discs and C3/4 posterior longitudinal ligament on T2-weighted images, with slight dura compression (Fig. 1b). The patient was treated with analgesics for 2 weeks, interrupted cervical traction for 2 weeks and cervical collar for 1 month. After a one-month conservative treatment, the patient’s symptoms greatly improved. VAS for cervical pain decreased to 1.0.

**Fig. 2:**
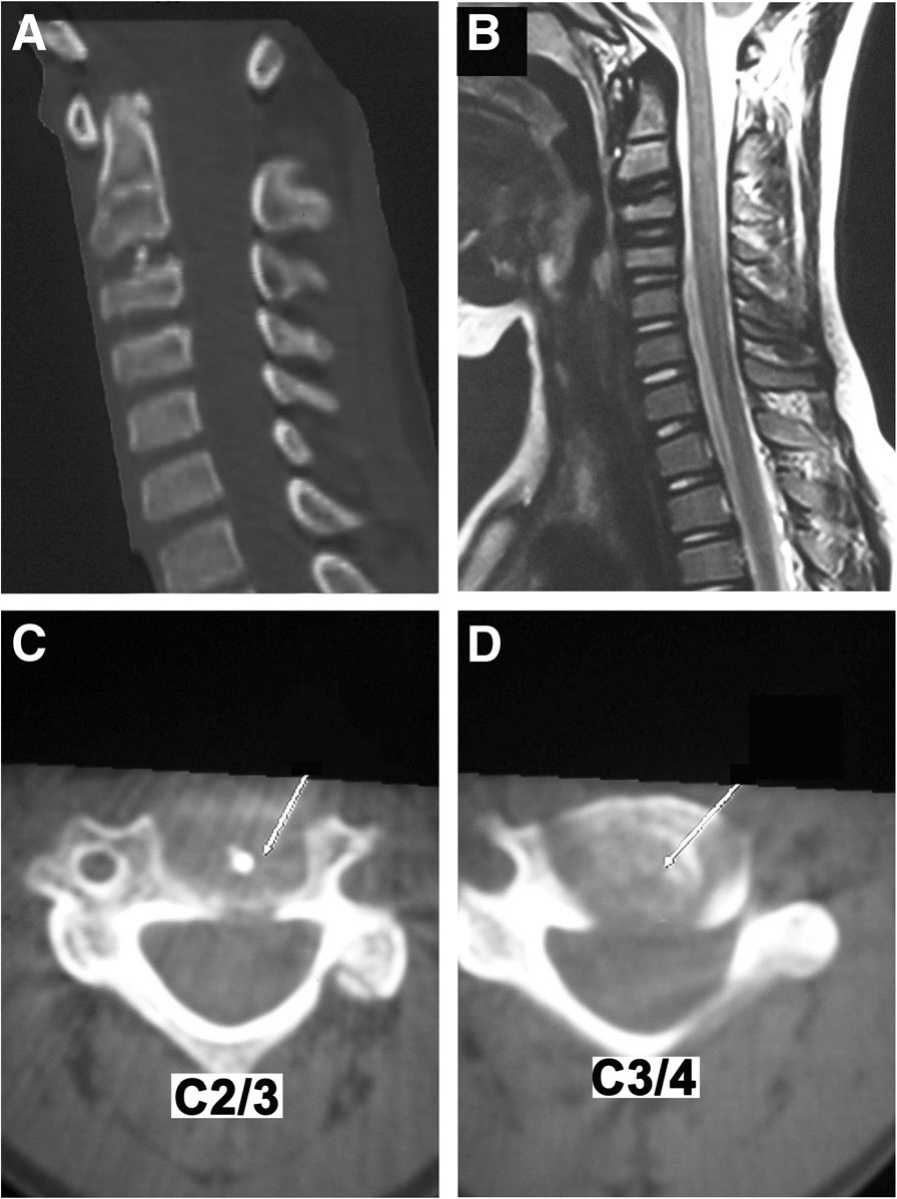
Radiological imaging at 19-month follow-up. **a** Computed tomography revealed the IDC and OPLL at the C3/4 level has disappeared, only minor calcification at C2/3 intervertebral disc left. **b** Magnetic resonance imaging revealed restoration of T2-weighted signal intensity of C2/3 and C3/4 discs. **c** Axial computed tomography through C2/3 revealed minor calcification at C2/3 intervertebral disc left. **d** Axial computed tomography through C3/4 revealed IDC and OPLL at the C3/4 level disappeared. (Adopted and reedited from Du et al. [3] with permissions of all authors)

Nineteen months later, in March 2009, the boy complained no discomfort. Laboratory tests (including white blood cell count, ESR and CRP) revealed nothing abnormal. C3/4 intervertebral disc calcification and OPLL had disappeared, only minor calcification at C2/3 intervertebral disc left (Fig. 2a, c and d). MRI demonstrated restoration of T2-weighted signal intensity of C2/3 and C3/4 discs (Fig. 2b).

**Fig. 3:**
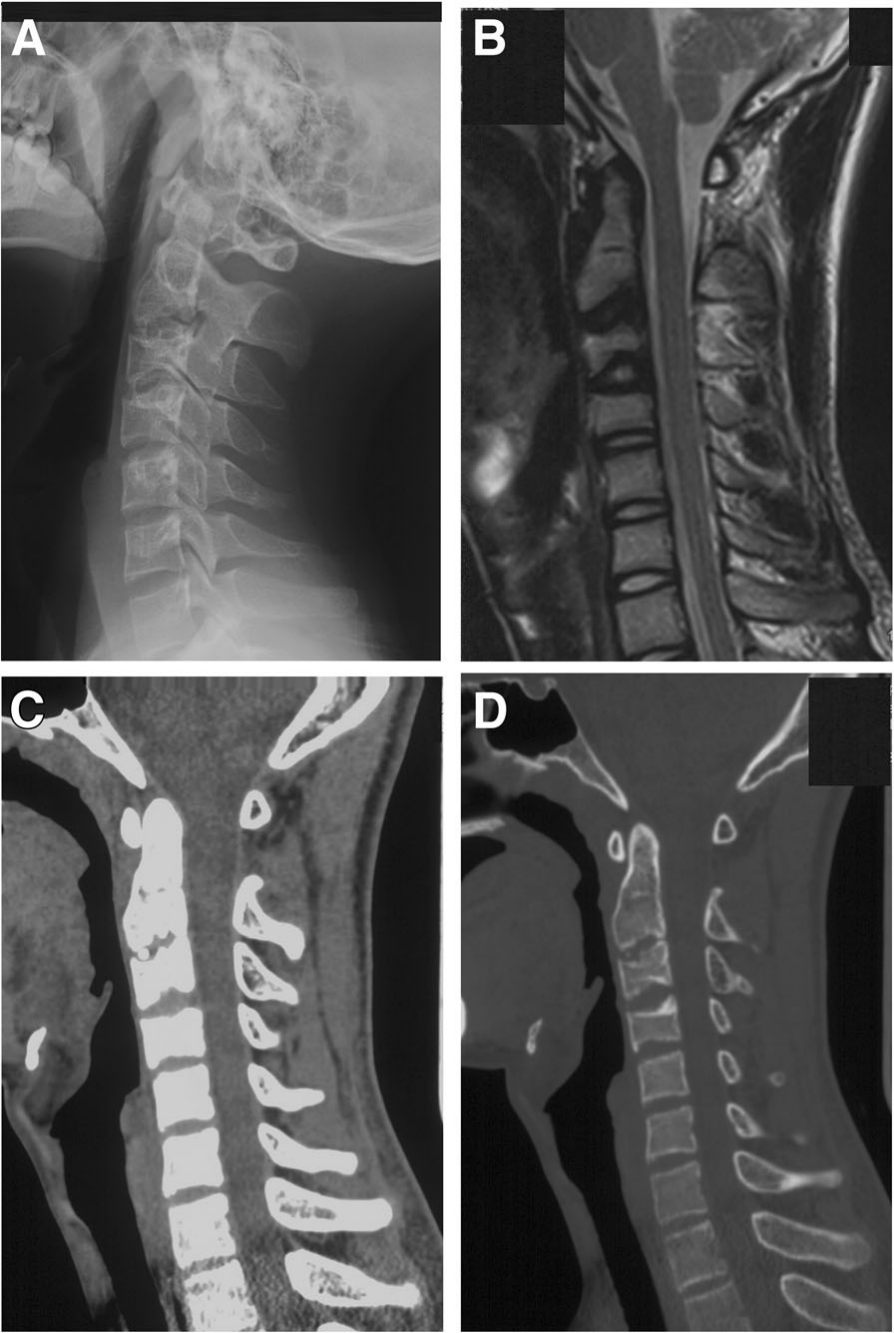
Radiological imaging at 9-year follow-up. **a** Lateral cervical spine radiograph showed minor IDC remained at C2/3. **b** Magnetic resonance imaging revealed loss of T2-weighted signal intensity of C2/3 disc and decrease of T2-weighted signal intensity of C3/4 disc. **c** and **d** Computed tomography revealed minor calcification at C2/3 intervertebral disc left, IDC and OPLL at the C3/4 level disappeared. Narrowing of C2/3 intervertebral space, flatting of C3 body, widening of posterior edge of C3/4 disc were observed

When last seen in October 2016, there was still no discomfort. Laboratory tests revealed nothing abnormal. No sign of C3/4 intervertebral disc calcification and OPLL was observed (Fig. 3a, c and d). Minor calcification at C2/3 intervertebral disc remained (Fig. 3a, c and d). MRI demonstrated loss of T2-weighted signal intensity of C2/3 disc and decrease of T2-weighted signal intensity of C3/4 disc (Fig. 3b). Narrowing of C2/3 intervertebral space, flatting of C3 body, widening of posterior edge of C3/4 disc were observed in CT scan (Fig. 3c and d).

## Discussion and conclusions

The incidence of intervertebral disc calcification (IDC) in children is low, with only approximately 400 cases reported since 1924. Intervertebral disc calcifications in children were divided in symptomatic and asymptomatic groups by Beluffi [7], who believed the number of asymptomatic patients could be larger than symptomatic patients. Blomquist et al. [8] reported 15 cases of IDC in children, of which 11 were symptomatic. Given that calcification of disc in children may be only an incidental finding without symptoms [9–12], the exact incidence may be underestimated [13, 14]. IDC mainly affect 5-to 12-year-old children [15], although newborn infant involvement was reported [7]. Males are more susceptible to IDC than females, with male-to-female ratio 13:6 [15, 16]. IDC mostly occurs in lower cervical spine and upper thoracic spine [17], with the most common level at C6/7 [18, 19]. First reported in 1838, OPLL has been widely reported since the 1960s [20]. OPLL usually affect people ages 50–70, the average onset age is 51.2 years in men and 48.9 years in women [21], with male-to-female ratio roughly 2:1 [22].OPLL is relatively more common in East Asian populations than Caucasian. The prevalence of OPLL was reported to be 1.5% to 3.7% in Japan and 0.1–1.7%in Europe and United States [21, 23–25]. The most commonly involved levels are C4–6 [26]. IDC with OPLL in children is an extremely rare situation, only six cases reported to date (Table 1) [2–6]. We reported the first two cases of cervical IDC with OPLL in children in 2012, while Fu et al. [2] reported the first thoracic case. Given that most reported cases occurred in East Asia, like OPLL in adult, IDC with OPLL in children may also have racial susceptibility.

**Table 1:** Reported cases of IDC combined with OPLL in children

Author	Reported year	Age/sex	Location	Pre-existing trauma	Clinical presentation	WBC (/mm^3^)/CRP (mg/L)/ESR (mm/h)	Radiographic changes/follow-up
Du et al. [3]	2012	8/F	IDC at C6/7 OPLL at C6/7	Yes	NP& ND	5860/16.5/55	IDC& OPLL disappeared/2 years
Fu et al. [2]	2011	11/M	IDC at T6/7,T7/8 OPLL atT6/7, T7	No	BP	Normal	IDC aggravated, OPLL alleviated/3 months
Wang et al. [4]	2016	11/F	IDC at C5/6 OPLL at C5/6, C6	No	NP	Normal	Mild IDC remained, OPLL disappeared/6 months
Mizukawa et al [5]	2017	6/F	IDC at C4/5 OPLL at C4/5	No	NP	8600/15/−	IDC& OPLL disappeared/6 months
O’Dell et al [6]	2016	9/M	IDC at C2/3 OPLL at C2/3	Yes	NP & stiffness torticollis;	–	IDC disappeared, mild OPLL remained/2 years
Current case	2012	6/M	IDC at C2/3, C3/4 OPLL atC3/4	No	NP	6170/11.8/69	Mild IDC remained, OPLL disappeared, /9 years

– not mentioned

*IDC* intervertebral disc calcification, *OPLL* ossification of posterior longitudinal ligament, *WBC* white blood cells, *CRP* C-reactive protein, *ESR* erythrocyte sedimentation rate, *NP* neck pain, *ND* neurological deficit, *BP* back pain

The etiology of IDC in children is still unclear. Trauma, infection, nutritional supply, vitamin D disorder, hereditary deficit may contribute to IDC in children [9, 13, 27–29]. Elevated ESR was reported to be the most sensitive indicator [15]. Coordinate with previous reports, elevated ESR and CRP are observed in our case, which suggested that infection may play a role in etiology of IDC in children. OPLL in adults is also considered to be multifactorial. Trauma [21], inflammation [30], genetics [31], environment [23], diet [32], glucose intolerance [33], obesity [33] and hypoparathyroidism [34] may contribute to the onset and progress of OPLL in adults. Trauma was seen in 2 cases of 6 reported cases of IDC with OPLL in children (incidence: 33.33%). Elevated inflammation indicators were seen in 3 cases (incidence: 50%, with one case didn’t give out inflammation indicators [6]). These results suggested that trauma and inflammation may play a role in the etiology of IDC with OPLL in children.

The most common clinical symptom of IDC in children is neck pain, affecting 80–90% cases [35]. Torticollis occurred in 40% of cases [11]. Other symptoms and signs include: perivertebral muscle spasms, low-grade fever, radicular pain, tenderness, and dysphagia (in anterior herniation cases). Only 5% patients of OPLL in adults were free of symptoms, 95% patients had clinical symptoms [21]. Different from IDC in children, varying degrees radiculopathy and myelopathy can be present in OPLL in adults [22], including balance dysfunction, muscular weakness, stagger, radicular pain, numbness and dysdiadochokinesia. Neck pain or back pain was seen in all the 6 reported cases of IDC with OPLL in children (incidence: 100%), neurological deficit (radicular pain), cervical stiffness, and torticollis was present in 1 case (incidence: 16.67%), respectively.

Conservative treatment, including analgesics, NSAIDS, muscle relaxants, cervical collar, traction and limited physical activity, is the mainstay treatment for IDC in children. Vast majority of children with IDC can be cure by conservative treatment. 66.7% patients got a complete relief of symptoms within 3 weeks and 95% patients would complete relieve within 6 months [19]. Recurrence of symptoms rarely occurs [36], but Hoffman [37] reported a child with IDC who suffered from neck pain and neurological deficit requiring surgery 6 years after initial diagnosis. Cases of IDC with symptom relapse 1 year after the initial onset were also reported [36]. Surgical treatment is controversial in cases with neurological deficit. Some authors suggested that conservative therapy could produce satisfactory results even when neurological deficit was present [9, 10]. Conservative treatment was proven effective even for the patient with neurological impairment due to large posterior protrusion [10]. Different from IDC with OPLL in children, surgery is more common for patients with OPLL in adults because of the progressive nature and poor prognosis [38]. Due to the extremely stenosis of cervical canal of the OPLL patients in adult, spinal cord injury (SCI) can occur even with minor trauma. Concerning that conservative treatments were adopted for all the 6 reported cases of IDC with OPLL in children with good effect, we suggest conservative treatment should be the first choice for these patients. Surgery should only be under consideration for cases with rapid progressive neurologic deterioration and high risk of paraplegia.

Coordinate with previous reports [16, 39–42], narrowing of the involved intervertebral space, flatting and wedging of adjacent vertebral body were observed in the current case at 9-year follow-up. IDC with OPLL in children seemed benign and self-limiting. Only mild IDC of C2/3remained but IDC of C3/4 and OPLL at C3/4 totally disappeared in the current case at 9-year follow-up. For all the 6 reported cases of IDC with OPLL in children, IDC disappeared in 3 cases (50%), aggravated in 1 case (16.67%), relieved but remained in 2 cases (33.33%). OPLL disappeared in 4 cases (66.67%), relieved but remained in 2 cases (33.33%). The only aggravated IDC case was treated by a 2-week lumbar belt immobilization [2]. Aggravation of IDC but relief of OPLL result in a reduction in spinal canal stenosis for the patient at 3-month follow-up, which made the conservative treatment still a promising choice. Given that this only reported aggravated IDC case was in thoracic disc, we can infer that thoracic IDC in children may have a different nature history with cervical IDC in children.

Through the 9-year follow-up, the changes of T2-weighted signal intensity for the involved discs drew our attention. Dehydration of intervertebral discs, which led to hypointense of T2-weighted signal intensity in MRI, was considered as a typical imaging manifestation of disc degeneration [43–45]. Restorations of T2-weighted signal intensity in MRI of degenerated discs were reported in several researches after dynamic stabilization systems implantation for low back pain patients, which were considered as decelerations of the degeneration process and regenerations of degenerated discs [46–49]. Nineteen months after initial diagnosis, restoration of T2-weighted signal intensity of C2/3 and C3/4 discs was observed in the current case through MRI. Similar change was reported by Liu [16], who reported a calcified disc restored to normal T2-weighted signal intensity at 2-year follow-up for a 10-year-old girl. The mechanisms of “rehydration” of the calcified discs are still unclear. Given that the spontaneous “rehydration” phenomenon is only seen in children but seldom adults, we can infer that this might be attribute to differences between discs of children and adults. The biggest differences between discs in children and in adults are the presence of microvascular blood supply for cartilage endplate and annulus fibrosus, as well as notochord cells, in children. Intervertebral discs appear vascularized more well in children than in adults [50]. Blood vessels penetrate into the anulus in infants but disappear by late childhood apart from some small capillaries [50–52]. The capillaries penetrate in the subchondral plate of intervertebral discs by regularly spaced nutrient canals in fetus and infants but disappear in childhood [52, 53]. The thickness of cartilaginous endplates of intervertebral discs diminishes with age [52, 54]. The notochordal cells exist in the intervertebral discs of fetus and infants but disappear by 10 years of age in humans, just as the time morphological signs of degeneration can be seen [55]. So, we speculated that these may contribute to the spontaneous “rehydration” phenomenon. Interestingly, similar “rehydration” phenomenon is seen in adult low back pain patients after dynamic stabilization systems implantation [46–49]. We can infer that the change of load distribution may also play a role in the “rehydration” phenomenon.

IDC with OPLL in children is very rare. Conservative treatments are recommended with affirmative short-term and long-term clinical effects. But given that such cases were so rare and radiographic changes in more than 30% cases didn’t improve, more intensive observation with long-term follow-ups may be needed to warrant the clinical effects.

## Abbreviations

CRP: C-reactive protein
CT: Computed tomography
ESR: Erythrocyte sedimentation rate
IDC: Intervertebral disc calcification
MRI: Magnetic resonance imaging
OPLL: Ossification of the posterior longitudinal ligament
WBC: White blood cells
